# A Secure Occupational Therapy Framework for Monitoring Cancer Patients’ Quality of Life †

**DOI:** 10.3390/s19235258

**Published:** 2019-11-29

**Authors:** Md. Abdur Rahman, Md. Mamunur Rashid, Julien Le Kernec, Bruno Philippe, Stuart J. Barnes, Francesco Fioranelli, Shufan Yang, Olivier Romain, Qammer H. Abbasi, George Loukas, Muhammad Imran

**Affiliations:** 1 Department of Cyber Security and Forensic Computing, College of Computer and Cyber Sciences (C3S), University of Prince Mugrin, Madinah 41499, Saudi Arabia; 2 Consumer and Organisational Digital Analytics (CODA) Research Centre, King’s Business School, King’s College, London WC2B 4BG, UK; mamun.rashid@kcl.ac.uk (M.M.R.); stuart.barnes@kcl.ac.uk (S.J.B.); 3 James Watt School of Engineering, University of Glasgow, Glasgow G12 8QQ, UK; Julien.LeKernec@glasgow.ac.uk (J.L.K.); Francesco.Fioranelli@glasgow.ac.uk (F.F.); Shufan.Yang@glasgow.ac.uk (S.Y.); Qammer.Abbasi@glasgow.ac.uk (Q.H.A.); Muhammad.Imran@glasgow.ac.uk (M.I.); 4 Laboratoire ETIS, Université Paris Seine, Université Cergy-Pontoise, ENSEA, CNRS, UMR8051, 95000 Paris, France; olivier.romain@u-cergy.fr; 5 School of Information and Communication, University of Electronic, Science, and Technology of China, Chengdu 610000, China; 6 Pneumology Department, René Dubos Hospital, 95300 Pontoise, France; bruno.philippe@ght-novo.fr; 7 Computing and Mathematical Sciences, University of Greenwich, London SE1 09LS, UK; G.Loukas@greenwich.ac.uk

**Keywords:** cyber-physical occupational therapy system, distributed medical big data, health IoT sensors, therapeutic kinematic data, quality of life

## Abstract

Once diagnosed with cancer, a patient goes through a series of diagnosis and tests, which are referred to as “after cancer treatment”. Due to the nature of the treatment and side effects, maintaining quality of life (QoL) in the home environment is a challenging task. Sometimes, a cancer patient’s situation changes abruptly as the functionality of certain organs deteriorates, which affects their QoL. One way of knowing the physiological functional status of a cancer patient is to design an occupational therapy. In this paper, we propose a blockchain and off-chain-based framework, which will allow multiple medical and ambient intelligent Internet of Things sensors to capture the QoL information from one’s home environment and securely share it with their community of interest. Using our proposed framework, both transactional records and multimedia big data can be shared with an oncologist or palliative care unit for real-time decision support. We have also developed blockchain-based data analytics, which will allow a clinician to visualize the immutable history of the patient’s data available from an in-home secure monitoring system for a better understanding of a patient’s current or historical states. Finally, we will present our current implementation status, which provides significant encouragement for further development.

## 1. Introduction

In 2018, 1.74 million individuals were diagnosed with cancer in the USA alone Both cancer itself and cancer treatments severely affect a patient’s quality of life and daily functioning [1,2], for example through muscle stiffness [3], joint pain, a decrease in bone density and muscle endurance, weight loss, nausea and vomiting, neurological impairments, decreased stamina, loss of range of motion, changes in sensation and cognition [4], cardiopulmonary dysfunction, deconditioning, difficulty with mobility and daily activities, edema, reproduction and sexuality difficulties, communication impairments, memory and attention problems, and psychological effects, such as depression, fear, anxiety, and post-traumatic stress disorder [2]. However, researchers have found that through early detection and advancements in treatment options, the survival rate can be improved [3,4]. Cancer patients go through severe hardship due to the nature of the treatment and its side effects [5]. Furthermore, since the advent of new targeted and more effective chemotherapy drugs, the patient’s care is more and more often ambulatory and prolonged. During the long-term management of cancer, the patient will initially be confronted with the mental load related to the diagnostic announcement, after which the patient will experience side effects from the treatments and variability of mood related to diagnostic announcements of remission or relapse. These items are of great concern to family and loved ones who are also facing a heavy mental burden that is often overlooked by caregivers. The severe side effects of cancer-treatment plans negatively affect the quality of life (QoL) and limit the body functionality of cancer patients [1]. Many of them experience difficulty in retaining their former physiological status, which usually impacts their activities of daily living (ADL), and returning to normal social, education, or work-related activities [6]. Childhood cancer survivors have reported hardship in conducting educational activities as per their potential, and adult cancer survivors with cancer-related cognitive dysfunction report problems with memory and concentration, as well as the ability to manage everyday responsibilities by him/herself, including self-care, taking care of dependents, participating in social activities, employment, and paying bills [7]. Unfortunately, a recent survey on practicing oncologist surgeons worldwide shows that 54.4% of the surgeons disclosed that they did not incorporate QoL data within their cancer treatment plans [8].

Physical activity is an important prognosis factor [9] in the outcome of cancer patients. Monitoring the incentive to physical activity can help improve QoL and survival prognosis of cancer. Additionally, adequate nutritional follow-up is necessary to meet energy needs, since both positive and negative energy balances are known to have deteriorating effects on cancer prognosis and mortality [10]. We argue that QoL monitoring can be achieved effectively, reliably, and securely by combining in-home Internet of Things with blockchain technologies in a manner that complements and learns from existing occupational therapy clinical practices (Section 1.1), patient data collection (Section 1.2), and follow-up care technologies (Section 1.3), as detailed below.

### 1.1. Cancer Follow-Up/Occupational Therapy

As a result of poor QoL support and increased levels of distress, health care facilities’ utilization and costs have increased [11], in addition to the lingering burden on caregiver family members. Prolonged pain, fatigue, difficulty in joint movement, and other side effects of treatment can be addressed with therapeutic rehabilitation services [11]. As a result, in addition to medical treatment, psychological, physical, and emotional consequences require a multidisciplinary team of supportive oncology care professionals such as an occupational therapist, physical therapist, speech and language therapist, hormone therapist, psychologist, nurses, and social workers. Under the premise of holistic care [12], the team can address the factors related to the patient’s ability to return to work [13,14].

In order to support a cancer patient, family members and caregivers often cannot fully concentrate on their professional work activities. Hence, a cancer patient’s functional disability has a social and economic impact. Unfortunately, cancer patients have been neglected by the existing advancement of cancer care when it comes to in-home supportive care for dealing with the psychological, physical, and emotional symptoms [12]. In one study, after breast cancer surgery, 50% of women reported a loss in range of motion (ROM) around the location of surgery [15]. Older adults who face daily fatigue need support in dealing with depression and pain. However, they are least likely to report the symptom to their practitioner due to a lack of such data collection framework at home and weakness in ADLs. Occupational therapy (OT) has the potential to increase participation in ADL, enhance quality of care, and improve QoL [16].

Existing research found that physical activity, which requires skeletal muscle movement and energy expenditure, leads to a better QoL for cancer patients [3,11]. Researchers have reported that increased physical and psychosocial function, which is addressed by OT, decreases the economic burden of cancer survivorship and engages the patient in life as independently as possible with the primary goal of improving QoL [12,13,17,18]. OT incorporates a variety of techniques and tools to propose ADL, control pain, and improve overall mental health, which are most important to one’s QoL [19,20,21]. State of the art OT techniques can support through assistive technologies, education regarding energy conservation techniques, anxiety management, guided relaxation, and key intervention [22]. Other psychosocial QoL parameters associated with impaired physical and emotional functioning include education about self-management, which enables making changes in the areas of cognition, behavior, and emotion to maintain the required level of QoL. OT can address these educational needs through the use of cognitive–behavioral approaches and interventions during routine care to address distress in cancer patients [23]. Creating secure evidence-based, cancer survivor-sourced OT interventions and techniques for the self-management of symptoms can revolutionize the existing cancer treatment landscape [18,24,25]. Functional limitations have an impact on the continuity of the full treatment cycle, as they are related to a higher possibility of co-morbidity and decreased survival [13,26,27]. 

Arranging a suitable environment that understands the needs of a vulnerable cancer patient at home is a challenging task [28]. The gradual increase in the number of cancer patients and a lack of the required number of oncologists and palliative care service providers have made the scenario even more difficult. Most of the time, practicing prescribed cancer therapies and maintaining a strict lifestyle is tedious and tiresome for patients, making it hard for cancer patients to maintain the proper quality of life [29]. Although sophisticated cancer or palliative care therapy facilities are available in cancer institutions, in-home cancer care is gaining popularity [30]. For example, due to the nature of after cancer treatment, certain types of cancer patients live with cancer for years, and cancer patients sometimes have to live in their natural home environment while the treatment continues. In the earlier stages, performing cancer therapy at home is more suitable for a patient, as it allows the assistance of caregiver family members. During the cancer treatment process, the immunization system of cancer patients weakens. As a result, the patients require more real-time interventions, and urgent follow up by the nursing or medical caregiver institutions [31]. Due to the high and abrupt level of side effects, it is important that the caregiver nurse, family member, or oncologist be in close touch with the patient’s physiological, neurological, or ambient states [32]. Although medical facilities such as palliative or intensive care units allow monitoring a cancer patient’s condition either intermittently or continuously, maintaining such a level of living lab environment at home for a very large population with cancer is becoming a societal need [33].

### 1.2. Cancer Patients’ QoL Data Collection

The objective of the OT for cancer care is the identification, gathering, and analysis of the needs, desires, and requirements of patient, carers, medical staff, and technology developers through a co-design approach. The traditional cancer care process starts by enrolling a patient at the cancer care institution; then, based on a diagnostic report, a follow-up protocol is defined for him/her, which includes the frequency of doctor visits. Patients are encouraged to perform OT addressing symptom assessments [34]. Since QoL data collection and symptom assessment are two different approaches and related to two different aspects of the disease, the QoL offers a translational assessment and reflects a chronic evaluation rather, while symptom assessment offers an alert of an acute health event (thoracic pain, syncope…). The assessment results from the OT session are auto-scored by algorithms, the results of QoL data are added to the patient’s electronic medical record (EMR), alerts are generated to notify clinical staff of critical patient condition, patients are provided with self-care instructions tailored to their needs, or the collection protocol may be changed based on the patient’s OT data [19]. Finally, the OT data can be shared with the national repository for further quality of care. The OT session data can be further used for the continuous improvement of cancer treatment to cope with the functional status of the patient. The OT-based subjective measures throughout different temporal points during the trajectory of the treatment can be used for informing individual patients, especially those who live in remote and rural areas distant from cancer centers, of how others have experienced the disease and treatments [35]. This would relax the stringent face-to-face meeting requirements with oncologists [36,37,38].

### 1.3. Technologies for Cancer Follow-Up Care

The above issues together are the drivers to develop state of the art cancer follow-up, for example, by varying the modes of delivering care (e.g., a serious game with an immersive OT environment), by allowing in-home cancer follow-up delivery (e.g., through an augmented-, virtual-, and mixed-reality-based telecollaboration environment) and by shifting the locus of care from hospital to the community [37]. In [39], a systematic overview of technology for cancer follow-up has been conducted. Given the potential penetration of digital technologies into our ADL, we envision future cancer research to embrace the futuristic models of next-generation cancer follow-up while employing security and privacy of the data, patient satisfaction and acceptability, and safety and cost-effectiveness. 

Mobile health technologies for OT interventions may enhance patient empowerment and data integration along with the whole cancer care ecosystem. However, these interventions pose relatively new regulatory, organizational, and technological challenges that limit appropriate evaluation [40]. The future development of cancer-care systems needs to incorporate patient-reported QoL data measurement as a feedback path to optimize the treatment plan. The review concluded that most systems focusing on pain reporting from patients were in the very early stages and calling for more automated development in the deployment of OT for cancer patients. It also pointed out that the reporting/communication was unilateral for the most part toward the clinicians, but that patients could also get much from feedback on their self-reported symptoms and improve standards of care.

Thanks to recent advancement in technologies such as medical Internet of Things (IoT), mobile edge/fog architecture, 5G communication, artificial intelligence (AI) for cancer data analytics, blockchain and off-chain for confidentiality, integrity, the availability of cancer patient’s data while in storage, transmission, processing or sharing states, to name a few, in-home cancer care is becoming affordable both technologically and cost-effectively [41]. Comparable to the ethical practices regarding how privacy and security permission is obtained from a patient or his/her caregiver family members, an in-house monitoring facility can be turned into a mini palliative care unit for monitoring quality of life [42]. Recent advancements in connected medical IoT devices have led to off-the-shelf electroencephalogram (EEG), electromyography (EMG), electrocardiogram (ECG), diabetic measurement, depression and anxiety management, and gait and movement tracking sensors are becoming ubiquitous [43]. Further, the digital big data analytics and AI-based symptom and semantic analysis of these medical IoT devices are becoming commonplace [44].

To maintain the pace of improvement, oncologists devise a treatment plan for each patient type, as the plan changes for different inter-patient and intra-patient cases [45]. An oncologist needs quality of life, pathological, and other types of relevant data to be able to adjust the plan accordingly. Hence, the in-home IoT devices can provide critical event and QoL information securely. Recent advancement in blockchain and off-chain technologies have shown encouraging prospects for storing key and salient cancer patients’ electronic health records (EHR) in the blockchain and electronic medical records (EMR) available from IoT devices in the off-chain decentralized repositories [46]. Since the amount of data generated by the IoT devices for such an in-home monitoring system is very large, the IoT data needs to be first captured by the local edge or fog network [47], processed locally at the fog nodes for security, privacy, and semantic data extraction, and then stored in the blockchain and remote off-chain big data repositories [48].

For example, EEG devices produce a quite large amount of motor imagery or somatosensory data. This also applies to non-invasive kinematic and kinetic gesture-tracking sensors, emotion-tracking sensors, and pathological IoT sensors [49]. Fog computing and mobile edge computing (MEC) is envisioned to allow the vast number of IoT devices to be handled locally. With the help of 5G device-to-device (D2D) non-licensed spectrum advancement, it is now possible to configure different IoT sensory data requiring different types of quality of experience, such as latency, jitter, and cache through cognitive radio network slices with the combination of a software-defined network (SDN) and network functions virtualization (NFV) [50]. The critical sensory data can be off-loaded to the nearby edge device [51] within the patient’s edge.

While technological advancement allows a vast range and type of quality of life data to be collected, applying privacy and security to the collected data is of the utmost importance [52]. Researchers have proposed many privacy, ethical, and security policies, which will allow in-home data collection for medical purposes. Moreover, technology such as Tor and blockchain allows data anonymity and confidentiality, respectively. Blockchain enables a cancer patient’s data confidentiality, data integrity, data ownership, data availability, and a secure data-sharing model. A patient or his/her caregiver can decide which data to share with whom and for how long [53]. This data sharing can be automated with the IoT lifecycle on the fly, which can be pre-programmed through a blockchain smart contract [54]. Smart contracts allow an oncologist to devise known treatment plans and prescribe a set of directions for the patient to follow. The smart contract can be activated upon spatiotemporal logic of the plan, with the support of blockchain data analytics and AI-based visualization. This will help different AI models of care that are appropriate for cancer patients with different illness trajectories. By looking at the trajectories, clinicians may plan personalized care to meet their patient’s QoL needs and help patients and caregivers cope with their particular situations [55].

In this paper, we propose a cancer patient’s in-home quality of life monitoring framework with the help of occupational therapy, which will allow an oncologist or a caregiver nurse to be securely in touch with the cancer patient under a treatment plan and follow up with day-to-day interventions. Our proposed framework will allow a number of quality of life tracking sensors—such as EEG, EMG, ECG, kinematic gesture tracking sensors, ADL tracking radar sensor, pupil tracking sensors, and ambient intelligent sensors—that reflect the quality and comfort of one’s physiology or surroundings to collect data that are part of the treatment framework and store these within the secure and private edge network that is allowed by the patient. The captured data (with key thresholds) are stored within the private blockchain network, while the raw sensory data is stored within the decentralized edge repository. The framework will allow a patient to decide which data to share with which community of interest and with what frequency. Our developed digital wallet for IoT devices, patients, oncologists, and other caregiver institutions will allow secure data sharing and visualization. We have developed distributed smartphone and web applications, which will allow the data to be captured, transmitted, and visualized by different stakeholders.

The remainder of the paper is organized as follows: Section 2 describes the literature review, while Section 3 presents system design. Section 4 illustrates the different aspects of scenarios that are supported by the framework and the implementation details. Finally, Section 5 shows the test results and discussion of therapy exercises performed in a smart home appliance control scenario, and we conclude the paper in Section 6. 

## 2. Literature Review

In this section, we will present our exhaustive literature review of the existing related works and suitability of OT for cancer care. The authors of [12] have shown a framework for occupational therapy practitioners in oncology to support both physical and psychosocial treatments for cancer survivors. The authors of [18] have surveyed the cancer treatment-related side effects such as cancer-related fatigue, cancer-related cognitive dysfunction, cancer-related peripheral neuropathy, cancer-related pain, lymphedema, and psychosocial issues that can be addressed by different domains of occupational therapy and provide a better quality of life. Reference [56] reports that patients who exercised after lung surgery had better fitness levels (measured using both a cycling test and the six-minute walk test) and strength in their leg muscles compared to those who did not exercise. Reference [56] also showed initial evidence for a better quality of life and less breathlessness in those who exercised. The authors of [57] have studied the 10 most prevalent cancer survivors in Norway to find out how many of them actually required occupational therapy and how many actually availed it. Although the need was mentioned, only 6% received occupational therapy. The authors of [13] did an extensive survey on relevant research evaluating the needs for occupational therapy for cancer survivors, the qualifying cancer-related conditions that can be addressed by occupational therapy, and potential interventions for occupational therapy. The authors identified many impairments related to cancer and its treatments that can be addressed through OT, such as cognitive function impairment, cancer-related fatigue, upper-extremity impairments, falls, and chemotherapy-induced peripheral neuropathy.

The authors of [16] developed OT sessions and implemented them on a cancer patient, which resulted in better QoL. The intervention consists of OT that would ensure energy conservation (tasks to lower energy demands, such as sitting while preparing meals), problem solving to aid with task analysis, education and problem solving to alleviate increased pain, serious games and strategies for reducing anxiety and depression over the loss of roles, strategies to explore sleep quality, yoga and expressive writing to decrease stress and improve quality of life, and home exercise containing range of motion and a stretching program to address fatigue. The authors of [22] outlined the OTs related to cancer rehabilitation and categorized them into seven areas: symptom control, activity training, patient education, motor training, sensory training, cognitive training, and vocational rehabilitation. The manuscript also recommended a set of OT cancer rehabilitation tasks and their evaluation instruments and assessment protocols. The authors found that OTs based on serious games should be tailored for childhood cancer survivors. 

The authors of [58] have shown how diversified types of OTs can improve cancer patients’ QoL by adjusting emotional, cognitive, and physical functioning, pain, and insomnia. The results were presented in the European Organization for Research and Treatment of Cancer (EORTC) Quality of Life Questionnaire (QLQ) scale. The authors of [15] showed the impacts of OT in oncology rehabilitation and the role of exercise in handling pain, fatigue, and musculoskeletal issues. The authors of [59] evaluated the OT on health-related quality of life (HRQoL) and engagement in ADL. The study found that women who had OT demonstrated statistically better scores in the global QoL, role functions, physical, emotional, cognitive, and social functions, fatigue, insomnia, diarrhea, constipation, appetite loss, nausea and vomiting, fatigue, financial impact, and other symptoms scales compared to the control group participants, as measured by the EORTC QLQ-C30 and its module EORTC QLQ-BR23. 

The authors of [60] measured the effectiveness of palliative care-based OT interventions. The authors investigated tools that met their inclusion criteria that can be evaluated for application by OTs for the treatment of cancer patients. The authors of [61] concluded that OT interventions support successful return-to-work assistance to cancer patients. The authors of [62] found evidence through randomized controlled trials that OT exercise interventions, whether the sessions were conducted for medium or longer time periods, considerably improved the QoL, social functioning, and physical functioning of cancer survivors. After studying 701 cancer patients, the authors reported that subjects with physical activity generally reported higher scores for most EORTC QLQ-C30 and Functional Assessment of Cancer Therapy: General (FACT-G) scales when compared to subjects without physical activity, indicating better QOL.

The authors of [23] tested the outcome measures such as time spent for self-management, adherence to prescribed medication, physical and psychological side effects, including QoL, on 76 patients. The authors have concluded that smartphone-based serious games can offer effective measures for patient QoL. The authors of [63] demonstrated the positive effects of tight communication and QoL data sharing between cancer patients going through treatment and the oncologist and other types of caregivers. The authors of [64] designed and implemented a mobile system for lung cancer patient follow-up. However, it lacks data privacy and security. 

Radar-based vital signs detection has been used for breathing disorder (BD) recognition and sleep stages (SS) [65,66]. In [67], radar is used first to recover individual heartbeats from radio frequency (RF) reflections, and subsequently, it uses the heartbeat sequence along with the breathing signal to recognize the person’s emotions such as sadness, anger, pleasure, and joy. The results shown for such a system are on par with ECG-based systems with 87% accuracy and 88.2% accuracy, respectively. The advantage of radar is that it is non-invasive and will not disrupt the patient’s day-to-day routine.

A large number of researchers have proposed access control-based approaches for managing health data. For example, the authors of [68] have proposed Context-Aware Access Control using Fuzzy logic (FCAAC), which combines a fuzzy model and ontology-based approach to express patient health-related contextual conditions that will allow healthcare providers in their decision making. On the other hand, FairAccess [69] offers a blockchain-based access control framework, which will allow the authorization of IoT data such as granting, obtaining, delegating, and revoking access. In another initiative [70], the access control of social network data has been designed to preserve privacy and trust. While the access control method has been implemented within social network data privacy and sharing, the access control mechanism can be leveraged for sharing the health data as an overlay layer on top of blockchain. A novel access control method of IoT data has been illustrated in [71] where the access control policies have been deployed as a smart contract within a blockchain. The framework also deploys a judge smart contract to monitor the behavior of IoT nodes based on the real-time data available from IoT nodes. Based on the smart contract policy and the types of data, the judge contract can either accept or punish the misbehavior of the IoT node. This will allow the fairness and validity of the IoT data, thereby adding explainability and reasoning to the IoT data. An interesting access control mechanism to support critical health-related events has been proposed in [72]. The system takes into account the dynamic contextual data available from a subject’s surrounding IoT data and maps that event into the management of critical situations. The authors have also introduced an ontology to map each dynamic contextual role with the associated access control policies. 

Although much advancement has taken place in the OT domain, sensor-based activity recognition, and data security, in-home QoL-enhancing OT interventions for cancer care treatment have yet to interweave the much-needed OT within the treatment plan. In this paper, we contribute toward bridging the gap by proposing a framework that takes advantage of technological advancements in IoT and blockchain-based data analytics to support OT-based QoL data as a complement to cancer care treatment. In the next section, we will propose the design of the OT system.

## 3. System Design

### 3.1. Preliminaries

In this section, we first deduce some preliminary design aspects and key software requirement specifications of an OT-based intervention that can be used within the existing cancer care protocols, instruments, and measurements. Cancer-related functional and cognition impairment takes place at a slow pace; hence, it is difficult to capture its entire spectrum during outpatient oncology office visits [13,73,74]. Hence, it is challenging to design OT screening tools. For example, geriatric assessment (GA) tool measures functional status, falls, and physical health cognition [13]. The patient-reported outcomes measurement information system (PROMIS) is a tool that assesses QoL, physiological status, and participation in social activities [13]. The disabilities of arm, shoulder, and hand (DASH) assessment tool is developed to assess the functional ability of the upper extremity [13], while the functional independence measure (FIM) tool can help in assessing the functional and mobility pattern. Among the existing OT-based interventions, SF-36 and EORTC-C30 were found to be most commonly used questionnaires by the oncologists [8]. After surveying lung cancer patients, the authors of [75] concluded that QoL data should be studied at every visit for each patient and in-between visits. Since OT is intended to allow ADL independently [76], OT QoL monitoring metrics such as the type, length, and frequency of therapeutic exercises, and change in the difficulty level or course of activities is recommended to be personalized [77,78,79,80,81,82,83,84,85,86,87,88,89]. Moreover, data privacy, confidentiality, and integrity can be assured by leveraging the recent advancement in blockchain and off-chain-based decentralized solutions, which guarantees the availability and scalability of OT data, proper end-to-end encryption, a digital wallet with secure cryptographic public/private keys, and high-speed transaction overlays [90,91].

### 3.2. Design Goals

**Figure 1:**
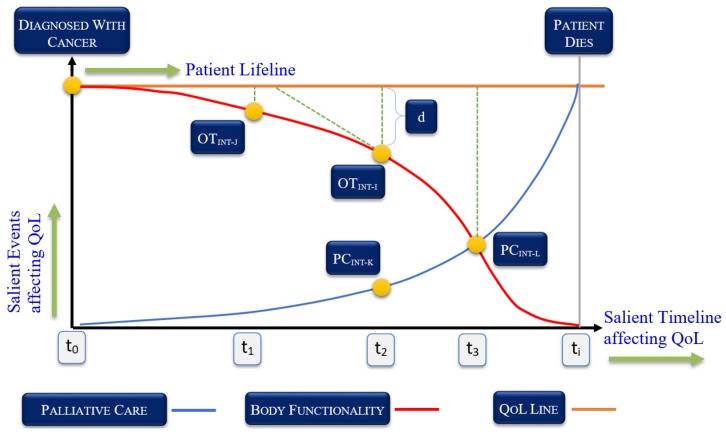
Relationship between quality of life and cancer treatment plan concerning different factors (such as occupational therapy) affecting the quality of life.

When a cancer patient is first diagnosed with a certain type of cancer, he/she immediately starts a treatment plan via an oncologist. Figure 1 shows the QoL management framework of a cancer patient in which some salient distinct timelines have been shown. Time t_0_ is the point at which cancer has been identified. The vertical axis shows the quality of life, which is expected to be maintained throughout the treatment process. However, depending on the type of cancer and the effectiveness of the treatment, the patient starts losing body functionality due to side effects or loss of immunity. The body functionality line represents how well the person feels at any given time. The farther this curve is from the quality of lifeline, the worse the patient feels. If the distance between the two lines increases significantly, the patient requires intervention, i.e., they are taken to the palliative care unit for maintaining their fundamental quality of life. The number of visits to the care unit and the length of stay there are decided by the oncologist. The effects of occupational therapy interventions (OT_INT_) are shown as a negative slope. The purpose of the OT is to keep the slope as close to the QoL line through customized OT interventions. The OT’s objective is to minimize the variable *d*, as shown in Figure 1. Another objective of the OT_INT_ is to minimize the palliative care intervention (PC_INT_) slope so that both the curves meet at the farthest temporal point in the care timeline ***t_i_***. 

### 3.3. Framework Design

As suggested by [22], the goal of our designed OT services are as follows:

- **Physical assistance**: The design goal is to provide physical support to manage symptoms such as fatigue or anxiety. The goal is to shift away from the support of a caregiver, and instead, the patient performs his/her daily activities as much as possible.
- **Supervision and hints**: The goal is to support a patient with several relevant OTs and design model therapies based on augmented and mixed reality.
- **Activity demands**: The task is that OT activities can be adapted based on the motor and cognitive demands of patients.
- **Sequencing of activity**: The role of this design is to discover the patient’s priority of activities through incentives. The number of steps in tasks, order, the frequency, and the complexities can be adjusted based on the QoL data while a cancer patient can complete the OT with motivation and fewer side effects.
- **Type of activity**: Depending on the functional state, the type of OT activities can be mapped with serious games and HRQoL instruments, thereby decreasing cancer-related symptoms.
- **Environment**: The OT activity environment tends to affect participation in ADL. Isolated environments such as palliative care make patients uncomfortable. Hence, to reduce symptoms and improve the performance of OT outcomes, it is imperative that the OT has to be designed for environments where a patient performs ADL, i.e., at home.

#### 3.3.1. OT Use Cases 

**Figure 2:**
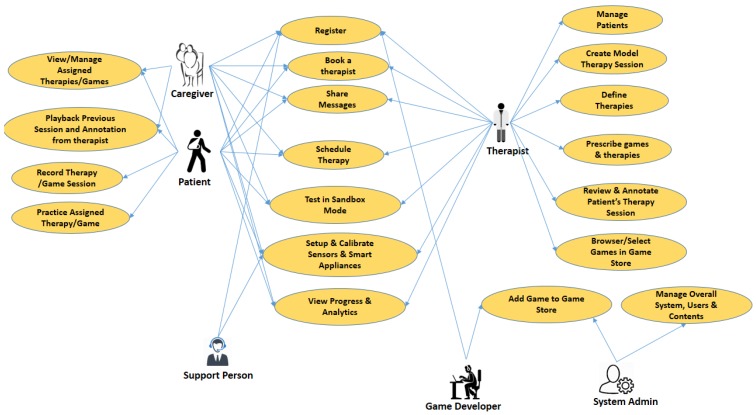
High-level occupational therapy use cases to assist in quality of life (QoL).

**Figure 3:**
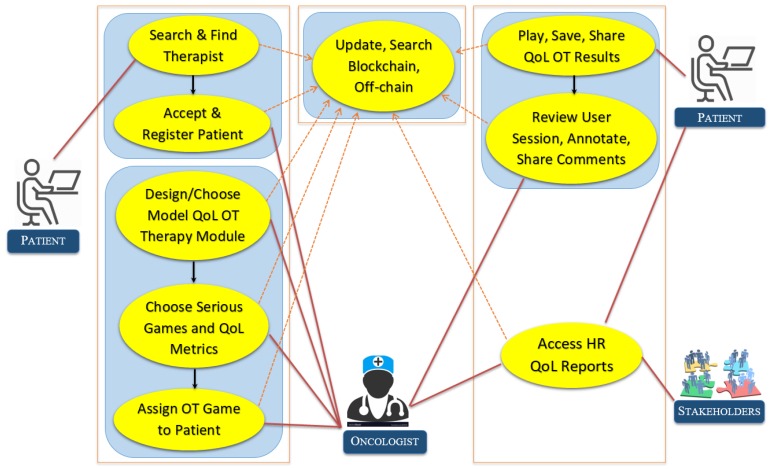
Therapist and patient can visualize the performance of the prescribed occupational therapy (OT) by looking at overall QoL data and functional status of the patient. HR QoL: health-related quality of life.

Figure 2 shows salient, high-level use cases of the system with different actors involved, while Figure 3 shows HRQoL-related use cases. The registration phase consists of two steps. In the first step, the patient or therapist will provide some basic information such as the patient’s name, mobile number, email address, and other necessary national ID. Their mobile number is verified with SMS, and the email address will also be verified. Finally, the profile data is stored in blockchain, and the uploaded files are stored in off-chain. After registration, a patient will be able to see the profiles of available therapists with expertise. The patient can write a secure message to a therapist and discuss his/her symptoms and disease with them. If a patient decided to pursue therapy from a therapist, the patient could send a request to the therapist. If the therapist agrees to take the patient, then the patient’s profile information will be visible to the therapist. Using the secure digital wallet, payment for an actual visit or online prescription, or secure location sharing or EHR profile sharing can also be done. Figure 3 shows a use case in which a therapist can create a therapy to suggest it to his patient. The therapist can further use it in the future, because all of the therapies he created are saved to his blockchain profile. He may also relate this therapy with a model session practiced by him. This way, patients will easily understand the ideal physical movement that has been prescribed for him/her. Once created and saved in the OT profile, a therapist can prescribe an existing therapy or game for the patient as well. Figure 3 shows the player design use cases as well. OT can be exercised in the following modes of operation.

- Sandbox-based practice exercises (session is not saved)
- Game-based therapy exercise
- Guided exercise through augmented reality view
- Guided exercise through telecollaboration view
- Guided exercise through a virtual reality view
- Guided exercise with robotic guidance
- Exercise through skeletal guidance

A therapist can play patient’s sessions and can annotate on it to provide multimedia feedback such as an online prescription and comments on the QoL data. Figure 3 shows different use cases of online spatiotemporal OT annotation. A therapist can provide an online comment on the OT session payload through the annotation player. The patient’s player can interact with the annotation marked on top of his/her OT session to adapt to the prescriptions and suggestions of the therapist. As shown in Figure 3, the patient can play to watch previously exercised sessions. If any session has an annotation on top of it, then it will be shown at the right temporal location. Figure 3 shows the use case of different visualization methodologies at a high level. 

#### 3.3.2. Software Components

**Figure 4:**
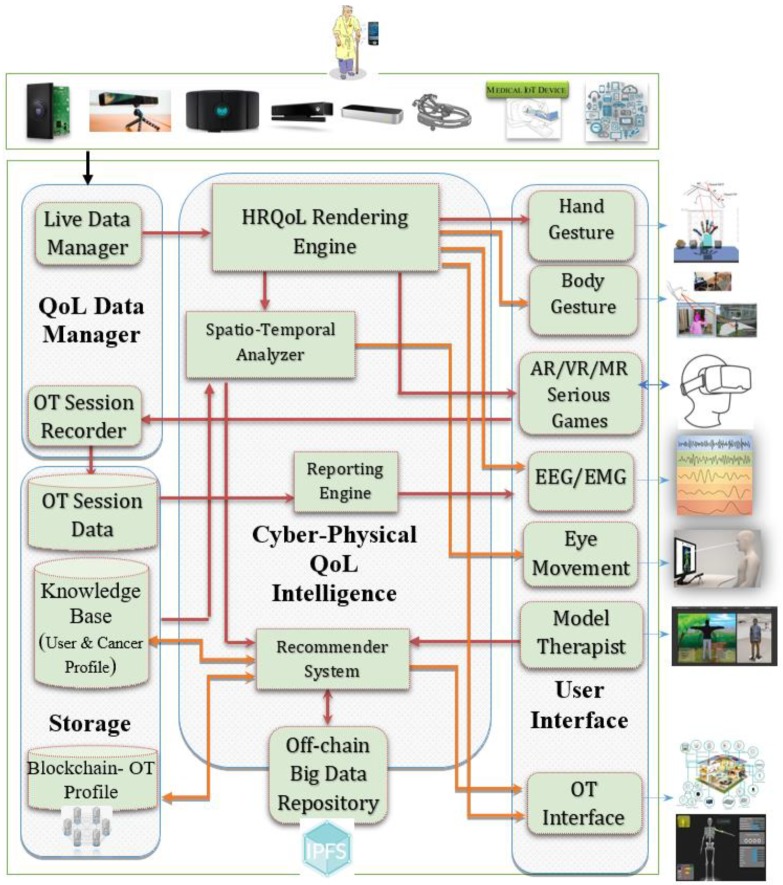
High-level software components of the occupational therapy environment to assist a cancer patient’s quality of life enhancement.

Figure 4 shows the high-level software components of the proposed framework. The QoL Data Manager is responsible for capturing the multisensory OT data and sharing with both live and off-line processing. One of its components, *Live Data Manager,* is responsible for live tracking the activities of daily living corresponding to OT, while the *OT Session Recorder* works while an active OT session takes place. The *Cyber-Physical QoL Intelligence* component houses all those components that process and intelligently recognize different OT actions. The *Health-Related QoL (HRQoL) Rendering* engine receives the streams of different sensory media, parses each stream, and based on the therapy, renders it to appropriate *User Interfaces*. The User Interfaces that require spatiotemporal processing, such as *Augmented Reality (AR), Virtual Reality (VR), and Mixed Reality (MR) Serious Games* [92], go through the spatiotemporal analyzer for adding OT game logic. The *User Interface* supports the visualization of OT or cancer-related QoL data in 2D, 3D, and IoT interfaces. The *Storage* comprises of *OT Session Data*, a *User Knowledge Base*, a *User/Therapy Profile*, and an immutable *Blockchain OT Profile* subcomponents. The *Reporting Engine* generates OT historical and improvement reports with different authorized stakeholders. Finally, the big data generated by sensory media or user-uploaded documents are stored at an *Off-Chain Big Data Repository*. 

#### 3.3.3. OT Sensing Platform

**Figure 5:**
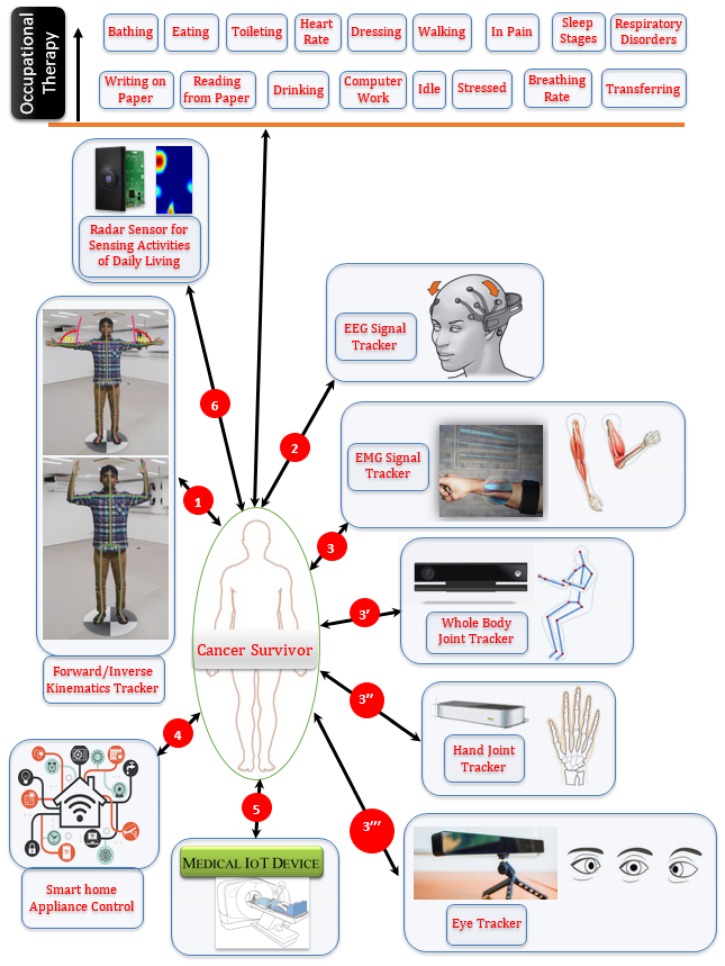
High-level sensory component diagram of the occupational therapy environment to assist cancer patient’s QoL.

Figure 5 shows an in-home OT sensing environment in which multiple sensors are assimilated to keep track of a patient. We have extended our existing research on brain–computer interface (BCI) [93] in which we investigated different aspects of EEG sensors that can provide sensory motor-based OT data support. Since this research only focuses on OT, we considered only beta and gamma frequency bands (13–100 Hz) captured from C3, C4, F7, and F8 electrodes, which carry motor imagery data in a sensorimotor rhythm. This also helps with filtering out a large volume of EEG data that are not relevant to the current research. Hence, only a subset of EEG data needs to be stored in blockchain for the QoL data analytics. We have also leveraged our prior work in handling EMG signals [41] and incorporated EMG signals within the framework, which can measure the well-being of muscle tones and the functional status of hand muscles. The range of motion (ROM) data is being tracked by Leap Motion and Microsoft Kinect V2 or HoloLens [94]. The pupil movement sensor provides gaze synchronization data, while the radar sensor offers different activities of daily life, as shown in Figure 5. 

The vital and other types of physiological data are available from other types of medical IoT devices. Smart home IoT appliances that help in daily chores are interfaced with the gesture-based natural user interfaces such as Leap Motion and Kinect V2. While interacting with the appliances for day-to-day life activity via gesture, the QoL data is being collected such as wellness of joints, muscles, movement accuracy, pain, distress, inertia, etc. Together, these multisensory environments can offer the semantic recognition of activities of daily life as part of OT for cancer patients. 

#### 3.3.4. Secure QoL Data-Sharing Architecture

**Figure 6:**
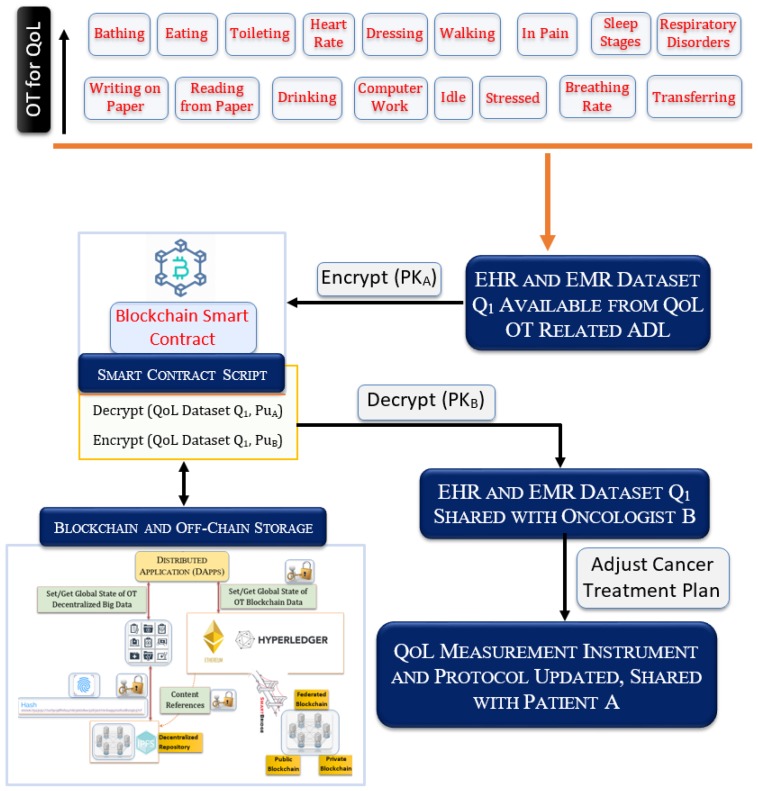
High-level blockchain and off-chain storage for securely sharing OT data.

Blockchain is attractive for applications where a set of distributed copies are required for redundancy [95,96,97]. In our proposed system, a cancer patient is monitored by a set of QoL-tracking ambient IoT nodes (see Figure 5). As shown in Figure 6, different types of OT data are made secure by the private/public keys. A patient can give access to his/her EHR and EMR data to a caregiver, therapist, insurance, doctor, hospital, etc. In addition, the patient’s data can be digitally signed and saved into blockchain by trusted parties. A patient can authorize a subset of his/her OT data on an ad hoc basis. A therapist can securely create smart contracts, which provide metadata related to a therapy that needs to be performed and save them for future usage. The smart contract also embeds the access policy of the patient. 

A patient or his/her family members can decide which data can be shared. For example, the data sharing can be instance-based, i.e., once only to a certain entity, several times, or with multiple parties at any given instance. Figure 6 shows a data-sharing model through the digital wallet, which will allow such data sharing. The EHR and the EMR data originated from OT are entered into the blockchain and off-chain after taking the public keys from the respective IoT devices, the hospital public key, the laboratory public key, and finally, that which is encrypted by the private key (PK_A_) of the patient. The patient intends to share a subset of historical as well as the QoL dataset available from his private blockchain, which is termed here as Q_1_. Hence, the system creates a smart contract to facilitate this transaction, which uses the public key of the patient (Pu_A_) to provide the decryption function Decrypt for access to the sharable dataset Q_1_. Once the smart contract has accessed the dataset Q_1_, it requests the public key of the oncologist (Pu_B_) to encrypt the QoL dataset Q_1_ and send a notification to the oncologist to visualize Q_1_. The oncologist can now decrypt the Q_1_ dataset by using his/her private key (PK_B_) from his/her digital wallet. After visualizing the QoL OT data, the therapist can decide the functional status of the patient and adjust the treatment. 

The QoL monitoring OT framework will result in a huge amount of data, e.g., text, image, audio, and video. Hence, off-line decentralized cloud storage can be used to store the data while the transaction in the blockchain stores the hash of the pointer or the files distributed in the cloud storage. While retrieving/querying a patient’s file, he/she must authorize with his/her private key, which is linked to his/her biometric profile, to obtain the hash of the distributed file pointers and then get hold of the actual file by providing the distributed hash to the cloud controller. The anonymity, security, immutability, integrity, and backup of the hash are guaranteed. To further the security and backup of the files, distributed, cryptographic peer-to-peer cloud storage architecture is adopted. Since the patient is in the center of the ownership of data stored in different autonomous and private health institutions’ computer systems, he/she can share his/her health data on demand through a blockchain-based smart contract.

**Figure 7:**
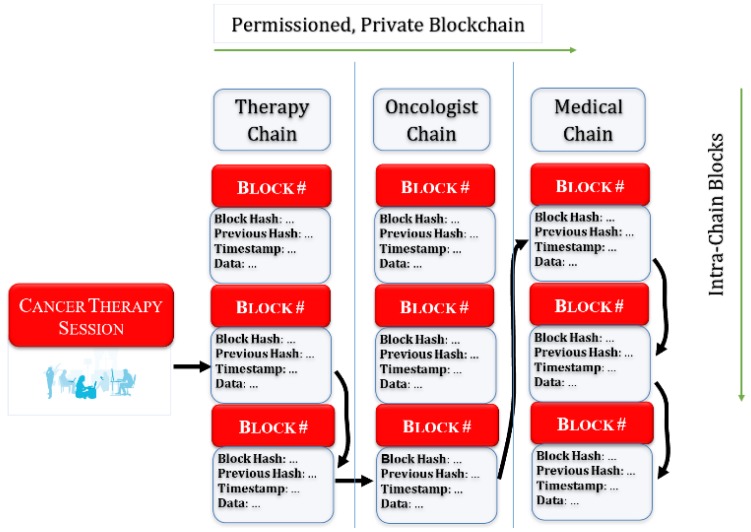
Example of a permissioned, multidimensional chain of a private blockchain to store cancer patients’ QoL data.

As a cancer patient goes through different stages of treatment, they are likely to suffer from side effects, co-morbidity, and/or the functionality of different organs may deteriorate. Hence, an oncologist may wish to refer a patient to a specialist for a particular organ or another clinical medicine expert. Figure 7 shows a sample scenario in which a patient visits different types of service providers, in addition to conducting OT activities at home. In each instance, a type of visit or test or OT session takes place, and the data generated from each visit is stored on a service provider’s permissioned, private blockchain, in addition to his/her own personal blockchain. This creates a multidimensional blockchain for each patient. Hence, a patient’s data is scattered in multiple private blockchains. Each vertical column represents the inter-patient blockchain, which holds all the test data of patients visiting a treatment facility.

#### 3.3.5. IoT-Based Natural User Interface Design for OT

Occupational therapy (OT) includes exercises to move the affected hand joints and muscles by performing daily life activities such as controlling a light, opening a door, driving a car, holding a pen, or holding a glass bottle, etc. OT measures how well a patient is doing in his/her daily life activity at home or outside of the therapist’s office. Unfortunately, in the absence of a physical therapist, the quality of improvement data of the patient remains undocumented. We have extended our previously designed gesture-based smart home appliance management system [98] by adding blockchain and off-chain, which also acts as an occupational therapy platform for a cancer patient. In our present architecture, the OT-related IoT appliances in a patient’s house are interconnected through a blockchain smart contract. Each gesture is interpreted as a specific OT action where each action has a QoL monitoring metric mapped to it. The gestures corresponding to QoL actions can be customized. When a cancer patient makes certain gestures in the OT environment, specific QoL data is collected. In our proposed research, off-the-shelf sensor devices such as Microsoft Kinect, the Leap Motion controller and the Myo EMG device from Thalmic labs, radar sensors, EEG, and other medical IoT hardware and serious games-based software-based frameworks have been investigated [99,100,101,102,103]. In our proposed system, we have combined smart home control with OT in a mixed reality environment using natural user gestures, which can be tailored for cancer patients’ QoL monitoring.

#### 3.3.6. Blockchain-Based Smart Contract Design for OT

**Figure 8:**
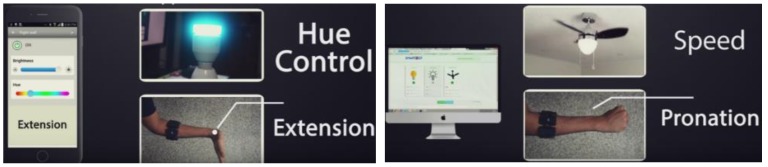
Occupation therapy exercise in a smart home appliance control scenario.

Understanding neurological, physiological, and psychological development after a person is identified with a certain type of cancer and arranging the most appropriate treatment plan that will either prevent cancer or prolong life after cancer is a demanding task [21]. Maintaining quality of life while the treatment takes place is a multifaceted problem: the definition of quality of life depends on user contexts, such as gender, socioeconomic situation, co-morbidity of diseases, types of the immune systems, geolocation, and caregiver support, to name a few. Modeling quality of life allows a computing system to map to certain quantifiable metrics and then to monitor those parameters [104]. The intelligent home environment can be customized and personalized for each type of individual cancer patient by coupling with OT. Figure 8 shows the OT environment in which IoT appliances can be controlled via gestures. 

**Figure 9:**
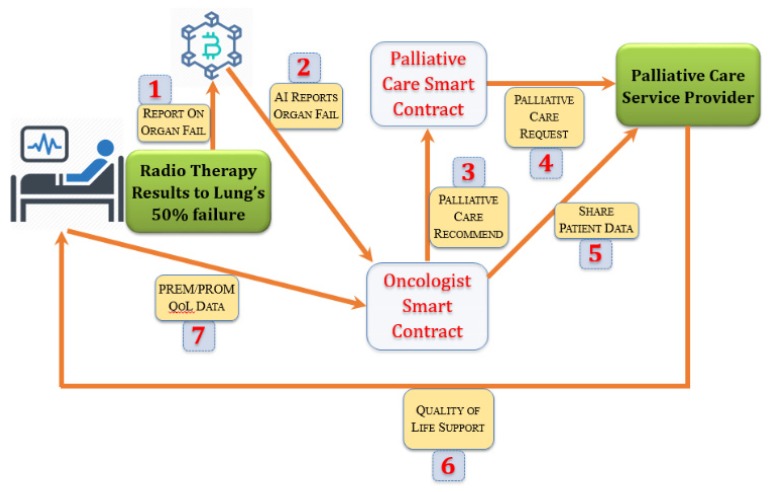
Salient components in a smart contract to support IoT-based quality of life within the home environment.

Figure 9 shows an automated process for monitoring a cancer patient’s functional status through a blockchain-based smart contract [105]. An oncologist can create a smart contract defining a set of rules that defines the functional well-being, which should trigger any status change within the treatment plan. The plan incorporates the quality of life data available from the ambient intelligent environment of the patient. As shown in Figure 9, in an instance of a patient, whenever radiotherapy results in a failure of a single side of a lung, this data can trigger the oncologist smart contract to activate a palliative care smart contract, so that the patient can be referred to a palliative care unit. While the patient receives care, quality of life can be deduced by the OT analytics, and the patient-reported outcome measures and patient-reported experience measures (PROMs and PREMs) data could be shared securely with the oncologist.

#### 3.3.7. QoL-Supported OT Therapy Design

**Figure 10:**
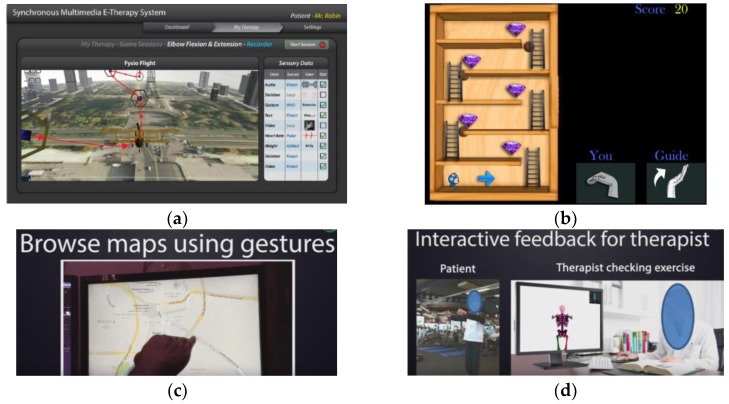
Game-based OT therapy exercise design: (**a**) gesture-based flying, (**b**) gesture-based jewel mining, (**c**) gesture-based map browsing, and (**d**) live skeletal data sharing with an authorized therapist.

**Figure 11:**
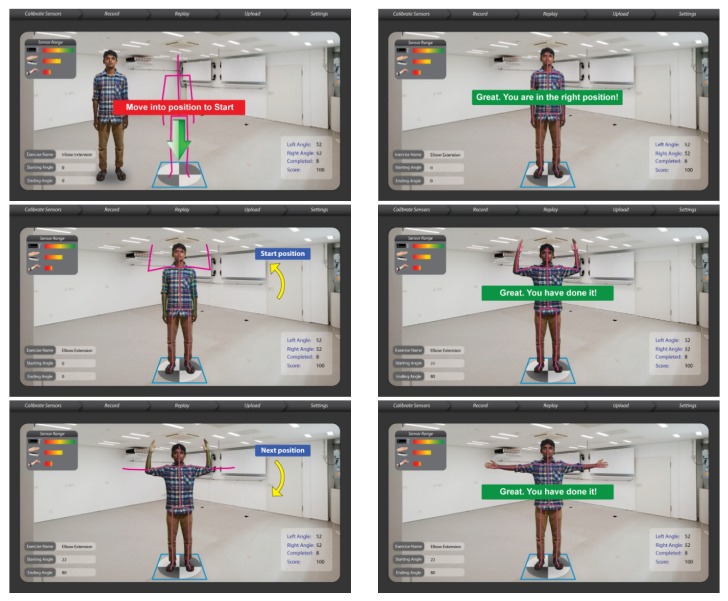
Guided OT exercise through augmented reality view.

**Figure 12:**
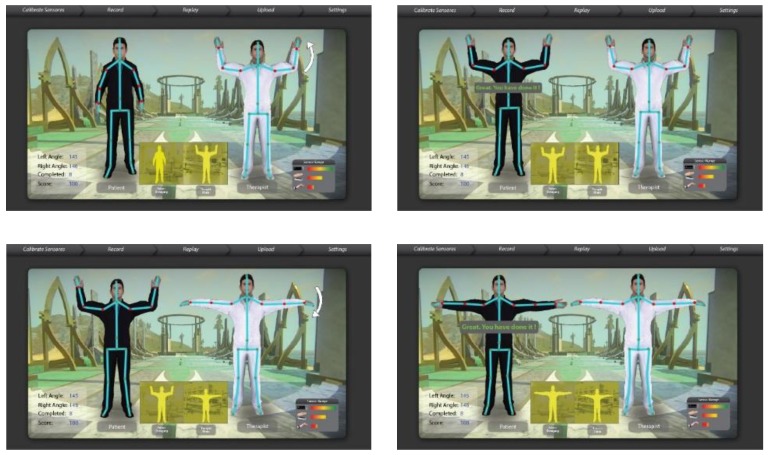
Guided OT Exercise through telecollaboration view.

**Figure 13:**
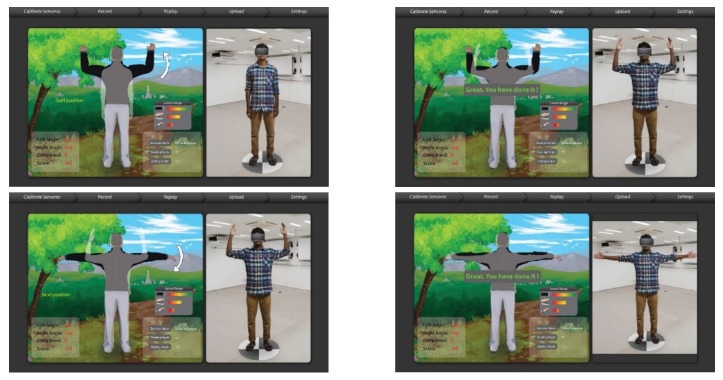
A therapist in the cyber world guides a subject in the physical world using a virtual reality view.

**Figure 14:**
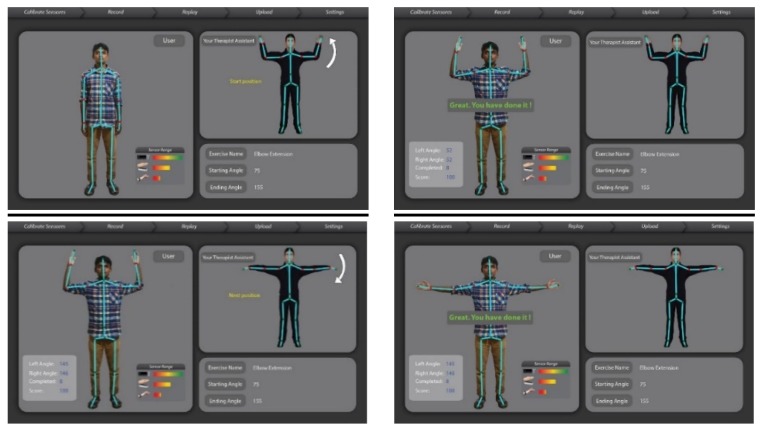
Virtual model therapist rendered as skeletal pose guides a subject.

**Figure 15:**
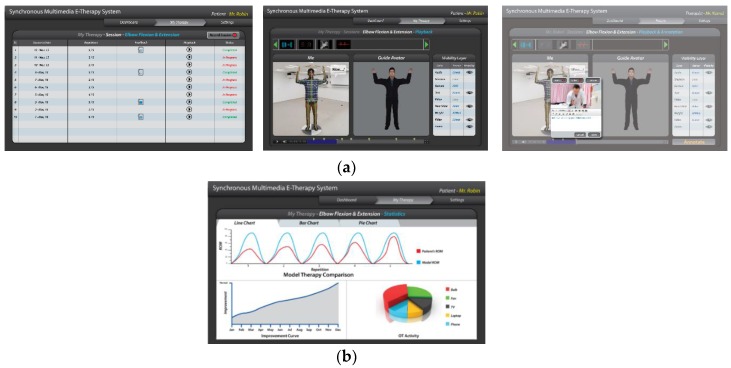
(**a**) Annotation window; and (**b**) analytic window.

**Figure 16:**
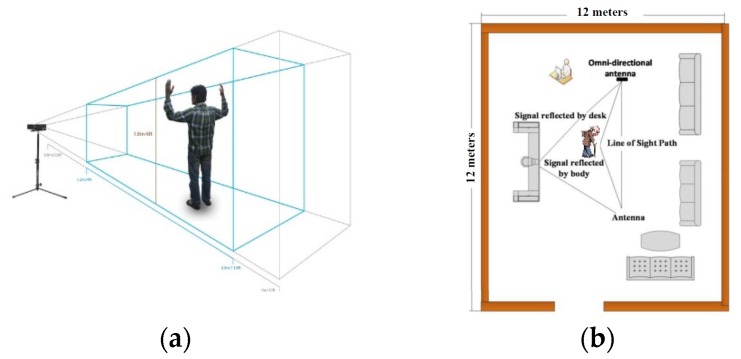
(**a**) Full body skeletal tracking sensor; and (**b**) RF sensing for assisted living—activity and vital sign monitoring.

**Figure 17:**
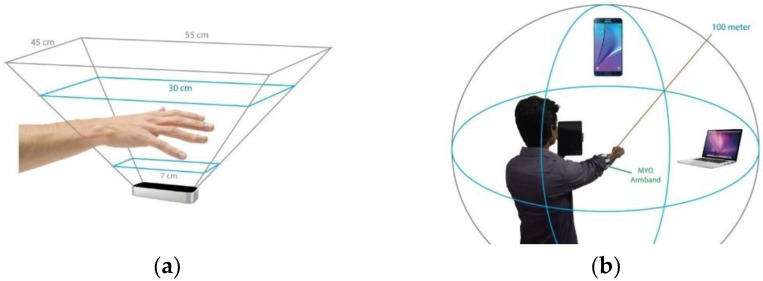
Hand skeletal tracking sensors (**a**) Spatial field of view of the LEAP motion sensor (**b**) Spatial field of view of the MYO EMG sensor.

**Figure 18:**
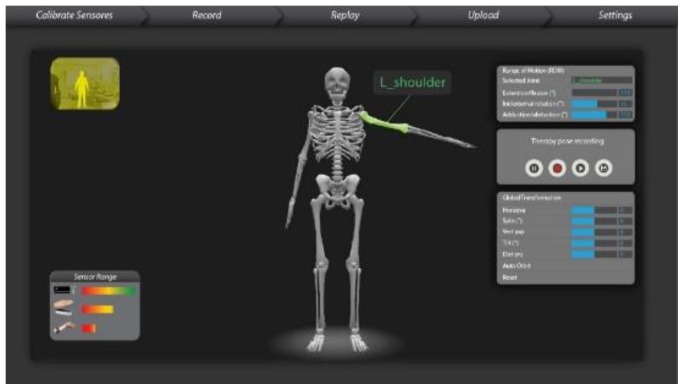
User interface of range of motion (ROM) BOT Digital Twin.

**Figure 19:**
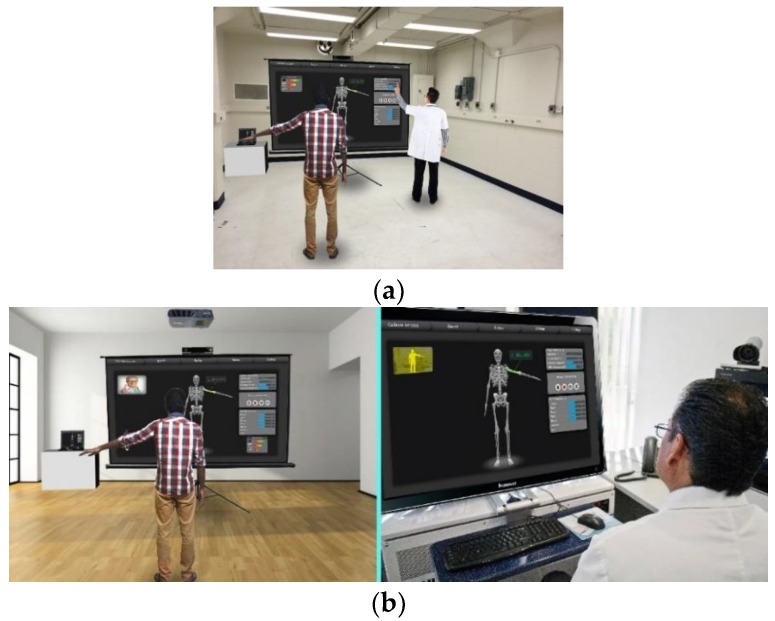
User Interface of ROM BOT Digital Twin, with the therapist either physically collocated or through remote collaboration: (**a**) both patient and the therapist are on the same physical proximity; (**b**) Therapist and the patient are at two separate locations but they can collaborate via the live sensory data sharing framework.

**Figure 20:**
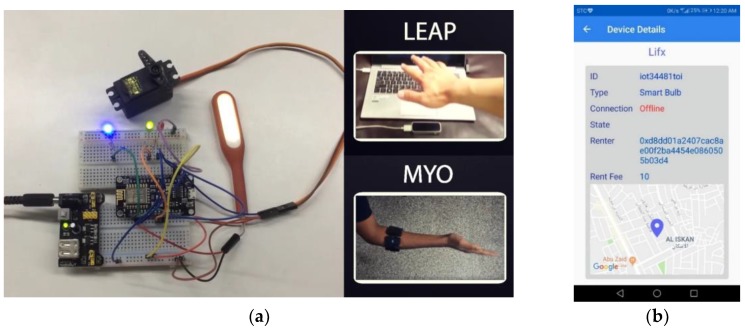
Implementation testbed: (**a**) Medical Internet of Things (IoT) devices connected to blockchain at the edge network; and (**b**) distributed application that securely shows the IoT device status extracted from the blockchain.

We have mapped the QoL metrics with those of some sample OT therapies so that the OT data become meaningful for the oncological treatment eco-system. We have taken into consideration the following QoL metrics: Assessment of QoL at End-of-Life (AQEL), Brief Hospice Inventory (BHI), Care-Notebook, City of Hope QoL Tool (CoH), Client Generated Index (CGI), EORTC QLQ-C30, Functional Assessment of Cancer Therapy—General (FACT-G); FACT-Pal, Functional Living Index—Cancer Scale (FLIC), Hospice Quality of Life Index—revised (HQLI), Life Evaluation Questionnaire (LEQ), McMaster Quality of Life Scale (MQLS), McGill Quality of Life Questionnaire (MQOL), Missoula-Vitas QoL Index (MVQoLI), Needs at the End-of-Life Screening Tool (NEST), *Palliative Care Assessment (PACA), Palliative Care Outcome Scale (POS), *Patient Evaluated Problem Score (PEPS), Quality of Life at the End-of-Life Measure (QUAL-E), *Quality of Life Scale-Cancer 2 (QOL-CA), Schedule for the Evaluation of Individual QoL (SEIQoL), SEIQoL—Direct Weighting (SEIQoL-DW), SF-36 Health Survey, Therapy Impact Questionnaire (TIQ), and WHOQOL-BREF. Figure 10, Figure 11, Figure 12, Figure 13, Figure 14, Figure 15, Figure 16, Figure 17, Figure 18, Figure 19 and Figure 20 show a model therapy guide for a subject for correctly performing an OT exercise via augmented reality, telecollaboration, virtual reality, and skeletal movement metaphors, respectively. 

**Figure 21:**
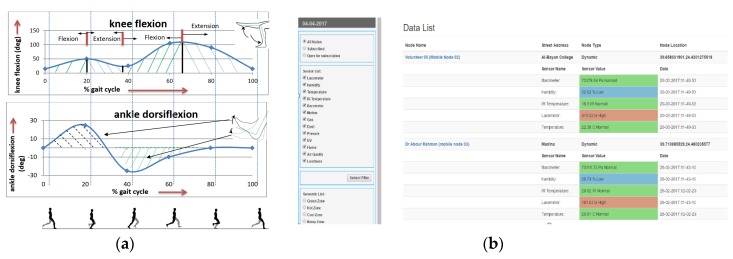
Quality-of-life data collected from sensory media and visualized from blockchain and off-chain data explorer: (**a**) live gait analysis to support occupational therapy and (**b**) semantic sensory data analytics.

Figure 21 shows an annotation option through which PROM data under the hood of OT sessions are captured, stored, indexed, and securely shared with the therapist. A therapist having edit privilege (as shown in Figure 6) opens a session, visualizes and plays the session temporally, and can annotate at any temporal point with multimedia. Figure 15 shows a sample analytics interface for a single patient. The analytics engine also supports comparative improvement statistics among a group of patients.

#### 3.3.8. Sensors and their Optimum Working Range

Figure 16 shows whole-body skeletal and activity tracking sensors and their field of view, while Figure 17 shows hand-tracking sensors. Similarly, we have incorporated EEG, EMG, and eye-tracking sensors, in addition to medical IoT sensors for heartbeat, Oxygen saturation (SpO2), glucose, weight, height, and other physiological data monitoring [65]. 

#### 3.3.9. ROM BOT—A Virtual 3D Digital Twin of Cancer Patient

The ROM BOT is a tool that provides solutions such as the real-time measurement of human skeletal ranges of motions and creating model OT exercises. An ROM BOT algorithm is crucial in guiding the OT sessions by patients, driving exercise game actions, and generating model therapy ROM, and thus helps with analytics and progress visualizations (see Figure 18). The following are some use cases of the ROM BOT for assisting in the OT of a cancer patient.

- A therapist can use ROM BOT to do an initial assessment of a patient’s overall or specific joint problem (see Figure 19).
- A therapist can use it to generate an ideal range of motion (ROM) of a specific joint or a set of joints, which can be customized into a set of model therapy steps.
- The engine that drives the ROM BOT comes in handy during the actual OT session by displaying a virtual skeleton that mimics the patient’s action with kinematic data.
- The ROM BOT can integrate with off-the-shelf skeletal tracking sensory products.

## 4. Implementation

In this research, we have developed a QoL framework for OT in which multiple sensory signals are used to detect activities of daily life and map the OT to capture QoL parameters. Based on the detected ADL, we design a smart Internet of Things (IoT) framework, which allows the patient to control home appliances such as lights, fans, AC, the oven timer, doors, etc., remotely using gestures similar to the real movement. The gestures can be configured such that each gesture is related to the disability level of the patient. Once the patient does any OT, the corresponding metrics of the therapy are recorded by the system. These metrics include the type and number of joints affected, the angle of rotation of each joint, the type and amount of motion produced, the number of times the patient successfully performed a physical activity, the time taken by that activity, and the minimum, maximum, average and standard deviation of the time and speed of performing these activities. Examples of these physical activities are opening a door using a knob rotating gesture, dimming a light using a particular gesture, speeding up a fan using a gesture, etc. When a patient performs OT such as turning the thermostat of the air-conditioner, the actual movement of the thermostat will take place on the smart switch connected to the network, and the patient will be able to see that on his terminal screen, too. Then, the OT movements are mapped to certain QoL measurement metrics or a questionnaire, depending on the therapy profile of a patient. Similarly, if the OT includes turning on a light switch, the light will turn on if the patient manages to perform the therapy correctly. In all cases, the user will be able to see the percentage of success of performing the therapy. The data generated by this OT session will give an occupational therapist as well as a physiotherapist insight into the improvement and effectiveness of the physiotherapy. 

To make this design more practical, the problem of device selection is also addressed. Suppose that a patient has several appliances in his/her room, which he/she wants to control. There should be a mechanism for deciding which appliance is in focus to know exactly the one to which the gestures should be directed. For this, we use the combination of pupil movement and EEG signal [106]. When a person looks toward a device, the system will be able to understand which device is intended based on the layout of the room stored in memory. This will solve the focus problem by selecting only a particular device, which is then controlled by a certain gesture. Another approach is displaying all the appliances on the screen of the viewer, irrespective of the direction of view of the user, using virtual reality (VR) technology. Finally, using our novel clinical data analytics, a therapist can visualize a live statistical analysis of the kinetic and kinematic motions and metrics and decide the quality of improvement of a particular patient clinically. The framework detects the following gestures that help in defining and measuring occupational therapy-related activities:

- Abduction/Adduction of all fingers and thumb
- Abduction/Adduction of a single finger
- Radial/Ulnar deviation around wrist joint
- Hyper-Flexion/Hyper-Extension around wrist joint
- Flexion/Extension around wrist joint
- Forearm pronation/supination
- Squeeze/Enlarge palm surface area
- Thumb touching middle finger

Figure 20 shows our developed non-invasive, quality of life-supporting ambient intelligent sensory board, which will allow different aspects of patient’s data to be captured. With the help of analytics and an AI model for each type of captured sensory data through the DApps, the data are securely stored at the patient’s edge network. We have tested an EEG device to capture somatosensory data, an EMG device to capture muscle tone, gesture-tracking sensors to capture kinematic and kinetic data, a pupil tracker for gaze tracking, medical IoT devices for vital information collection, and smart home IoT devices to collect user ambient information. Figure 20 shows the ambient intelligent environment surrounding a cancer patient, employing a set of IoT sensors to be able to capture quality of life-related data, digesting the captured data ubiquitously, saving it to the nearby edge blockchain or decentralized big data repository, using deep learning AI techniques to find phenomena of interest, and applying monitoring analytics to share the right data with the community of interest. While the transactions are stored in the blockchain, the EMR data will be stored in the off-chain, while the hashes of the off-chain will be stored in the block for further references. The data that need to be shared with a different community of interest can be accessed through our developed distributed applications (DApps). For example, we have developed DApps for patients, family members, cancer therapists, a secure online doctor on demand, hospital personnel, and medical IoT devices to be able to record the data of cancer patients, caregivers to be able to be in touch with the patients, and data management planning. Figure 20a shows the IoT hardware implementation setup in which a mobile edge network includes a set of IoT devices in diverse categories, such as an LIFX light bulb, door lock, Emotiv EEG device, MYO EMG device, LEAP motion hand gesture tracking sensor, Kinect for Windows V2 body gesture tracking sensor, and the Eye Tribe pupil tracking sensor, which are all interfaced with the cyber-physical system. The sensors can update the captured data through the secure digital wallet designed for a patient’s quality of life digital analytics, as shown in Figure 20b. The DApp shows different lifecycles of interaction with different IoT devices and the data obtained from these devices.

Figure 21 shows the different quality-of-life sensory data after adding semantic values through our developed data analytics [107]. The developed DApps captures the sensory data, applies the privacy and encryption model through the digital wallet parameters, and offloads the data to the edge network for further data storage and sharing. The Blockchain explorer shown in Figure 7 groups together sensory media to apply semantic rules. For example, a subset of sensory data can be grouped to deduce the quality of life context, such as “feeling comfortable”, “feeling suffocated”, “feeling exhausted”, “could not sleep at night”, “did not go to washroom for a day”, “forgot to take the medication”, and so on.

## 5. Discussion of Test Results

### 5.1. Continuous Improvement through User Co-Design 

We have worked with therapists from four hospitals in Makkah, which are specialized in patients needing OT. The therapists offered to help tailor our proposed OT framework to their real patients and test serious games-based modules that were the most relevant to them. In the first case, the kids in the Disabled Children Association (DCA) hospital were highly immersed within the OT environment. The therapists showed positive response with the usefulness and performance of our framework. While working with the therapists and actual patients, we are on a path of continuous improvement. We have been adjusting different security aspects within the framework. However, we could not test some modules, such as mixed reality, and remote telecollaboration-based therapy modules, due to a long process of obtaining approval from different entities. In the near future, we are planning to test these modules on real patients to find their efficacy in the therapy domain. Another important aspect that requires more design attention is to include a business model for therapists to agree in a telecollaboration session. We are working with therapists to overcome these non-technical challenges.

### 5.2. Blockchain Performance Testing 

We have tested the blockchain machine farm with a physical setup consisting of 4 Core, 8 threads, and 16 GB of RAM. The IoT data-sending rate was maintained at 300 transactions per second (tps). The following table shows the latency metadata of the test. Table 1 shows the summary of the system benchmark test, in which the maximum latency is 4.32 seconds, the minimum latency is 1 second, and the average latency is 2.9 seconds for a benchmark test of 266 transactions per second. The latency is because the high throughput IoT data needs to be captured, parsed, and then sent to the private, permissioned blockchain nodes, which approve the transactions and add the InterPlanetary File System (IPFS) hash to the blockchain.

Table 1 assumes different blockchain and control nodes with a certificate authority that are part of the blockchain and off-chain clients. The inbound and outbound traffic data, the CPU engagement, and the memory used during the benchmark test show that the framework could handle a large number of concurrent traffic. However, in the future, we will be working on scaling the framework through multiplexing and using other non-orthogonal blockchain frameworks.

**Figure 22:**
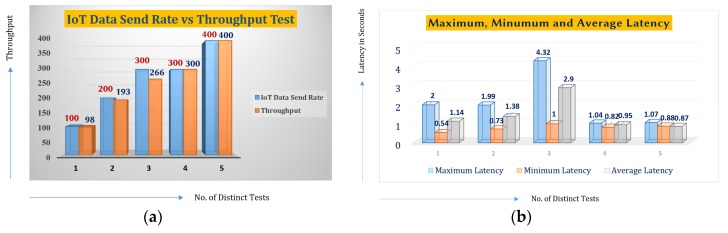
(**a**) Testing based on 5000 transactions made on five different instances of throughput measurement, and (**b**) testing based on 5000 transactions made on five different instances of latency measurement.

Figure 22a,b show the test results in which we carried out five instances of OT transactions in different test conditions and scenarios. In each test instance, 5000 transactions were made. In each transaction, IoT data from IoT devices were either added to the blockchain or read queries were executed. Test instances 1, 2, and 3 show some packet losses, while instances 4 and 5 show no packet loss. We assume that this packet loss happens due to network connectivity issues. In the future, we will investigate the cause and try to improve system efficiency. As for the delay, the current load-testing outcome is due to the current algorithmic shortcomings of the consensus algorithms of blockchain. However, 5000 transactions per second seem tolerable for OT applications. In the near future, we will investigate how to lower the latency by benefitting from the non-orthogonal frequency division multiple access and other innovations introduced by 5G communications.

## 6. Conclusions

In this paper, we have presented a framework that can support QoL metrics during cancer care treatment. The occupational therapy system mashes up multiple sensors for capturing activities of daily life through a game-based non-invasive environment. The framework aims to map the joint motions and gestures as they occur in the physical human body through an appropriate avatar in the 3D cyber world and then produces necessary metrics that facilitate therapeutic decision-making for a specific patient. The framework supports therapy recording, playback, and semantic annotation of a therapy session through a blockchain-based immutable environment. Through our proposed secure QoL monitoring framework, a virtual model therapist can guide a patient using a recorded model therapy session through skeletal animation, augmented reality, and virtual reality view. In addition, if a patient requests a live telesession to get an online and instantaneous comment from the therapist, he/she can establish a live therapy session where the privacy of both is protected.

Moreover, the framework is suitable for distracted subjects and occupation therapy exercise disguised under a smart home appliance control application. The framework uses the potential of decentralized big data technologies to assist in secure QoL data sharing. Our developed multisensory IoT-based framework is specifically tailored for the quality of life tracking of cancer patients. To preserve the privacy, confidentiality, data integrity, and data availability of edge storage, during transmission and processing, we have introduced a blockchain and off-chain-based decentralized framework. We have developed analytics to interact with the blockchain and off-chain for the semantic retrieval of quality-of-life-related data. We have also developed a secure EHR and EMR data-sharing algorithm, which will allow a patient to securely share a subset of his/her health data with a subset of the community of interest. In the future, we are planning to work with clinicians and hospitals to do clinical trials for real-life data collection and to assess the suitability of the framework for widespread usage. This work paves the way for new approaches for monitoring a cancer patient’s quality of life remotely and in near real-time, thus contributing to spotting the quality-of-life deterioration much more quickly than with traditional approaches.

## Figures and Tables

**Table 1:** Latency testing for OT data storage to blockchain benchmarks.

Max Latency	Min Latency	Avg. Latency	Throughput
4.32 s	1s	2.9s	266 tps
